# Factors influencing postoperative length of stay in an enhanced recovery after surgery program for primary total knee arthroplasty

**DOI:** 10.1186/s13018-018-0729-x

**Published:** 2018-02-02

**Authors:** Shaoyun Zhang, Qiang Huang, Jinwei Xie, Bin Xu, Guorui Cao, Fuxing Pei

**Affiliations:** Department of Orthopaedics, West China Hospital, Sichuan University, Chengdu, Sichuan 610041 China

**Keywords:** Total knee arthroplasty, Enhanced recovery after surgery, Postoperative length of stay, Influencing factors, Postoperative complications

## Abstract

**Background:**

Hospital length of stay (LOS) after primary total knee arthroplasty (TKA) has decreased obviously following the implementation of enhanced recovery after surgery (ERAS) program in the last few years. However, there are still some patients that cannot be discharged at early time for a variety of reasons, and it is necessary to explore factors leading to prolonged LOS. Therefore, the purpose of this study was to identify the complete preoperative, perioperative, and postoperative factors associated with prolonged postoperative LOS (PLOS) after primary TKA in a detailed ERAS program.

**Methods:**

In a consecutive series from July 2015 to March 2017, all patients who underwent unilateral elective primary TKA were included in the retrospective study. A PLOS greater than 3 days was considered a prolonged PLOS. Multivariable logistic regression analysis was performed to identify patient characteristics and relevant preoperative, perioperative, and postoperative variables that were associated with prolonged PLOS and postoperative complications.

**Results:**

A total of 241 patients were included with a mean PLOS of 3.8 days. Prolonged PLOS was significantly associated with preoperative valgus deformity of the knee (OR 4.95, 95%CI 1.56–15.77, *P* = 0.007), increased serum level of interleukin-6 on postoperative day 1 (OR 1.01, 95%CI 1.00–1.03, *P* = 0.039), increased visual analogue scale pain score and serum level of C-reactive protein on postoperative day 3 (OR 2.56, 95%CI 1.28–5.13, *P* = 0.008; OR 1.01, 95%CI 1.00–1.03, *P* = 0.019), increased day to achieve 90° active knee flexion after surgery (OR 2.19, 95%CI 1.27–3.79, *P* = 0.005), and postoperative wound complications (OR 8.58, 95%CI 2.10–35.03, *P* = 0.003) and other minor complications (OR 6.04, 95%CI 2.40–15.19, *P* < 0.001). Preoperative pulmonary infection (OR 2.75, 95%CI 1.20–6.28, *P* = 0.016), American Society of Anesthesiologists score 3/4 (OR 2.14, 95%CI 1.01–4.52, *P* = 0.046), and utilization of catheter after surgery (OR 2.53, 95%CI 1.23–5.19, *P* = 0.012) were significantly associated with postoperative complications.

**Conclusions:**

Multiple factors were associated with prolonged PLOS and postoperative complications after TKA in the ERAS program. It is important to recognize all the factors to try to maximize the use of medical resources and ultimately optimize the care of our patients.

## Background

In the last few years, hospital length of stay (LOS) after primary total knee arthroplasty (TKA) has decreased following the implementation of enhanced recovery after surgery (ERAS) program [1, 2]. This ERAS program is a series of perioperative multimodal strategies to enhance recovery and reduce morbidity [1]. Enhanced-recovery TKA has been shown to be safe and feasible, with similar or better outcomes for the patients [2, 3].

In literature, although the postoperative LOS (PLOS) of TKA has decreased substantially, there are still some risk factors that influence PLOS including older age [4–7], female [5–7], increased body mass index (BMI) [4, 8], American Society of Anesthesiologists (ASA) score 3/4 [5, 6], surgery on Thursday or Friday [6, 9], not ambulating on day of surgery [10–12], and postoperative complications [7, 11]. However, some of those studies have investigated prolonged PLOS in the absence of an ERAS program [5, 7], focused only on preoperative patient characteristics [4], not mentioning the definition of prolonged PLOS [4, 9, 11]. Furthermore, factors such as pain level, preoperative knee function, perioperative laboratory examination, surgical factors, and postoperative complications were often rarely mentioned.

The ERAS program of TKA in our hospital was also introduced with the aim to reduce PLOS to a mean of 3 days with equal or higher quality of care. To find starting points for further reduction of the PLOS in the future, we studied a consecutive series of 241 patients admitted for primary TKA to identify all risk factors for prolonged PLOS in combination with the ERAS program. Specifically, the relationships between PLOS, patient demographics, comorbidities, surgical factors, and postoperative complications were assessed. It was hypothesized that several preoperative and postoperative variables are factors that influence PLOS after TKA, such as comorbidities, preoperative knee function, pain level, and postoperative complications.

## Methods

We retrospectively reviewed a consecutive series of 241 patients who underwent unilateral elective primary TKA from July 2015 to March 2017 in our hospital. Institutional review board approval was obtained. The primary outcome of the study was PLOS, defined as the number of nights from surgery to discharge because all patients were admitted to the hospital several days before the surgery. Since the standard target for discharge in our practice was postoperative day 3, prolonged hospitalization was defined as PLOS > 3. There were 123 (51.0%) patients who had a PLOS ≤ 3 days and 118 (49.0%) a PLOS > 3 days. All surgeries were performed in an ERAS program and by one experienced surgeon performing > 100 TKAs per year.

### ERAS program

All patients had access to the full ERAS program, which included preoperative, perioperative, and postoperative protocols for the delivery of care (Table 1).

**Table 1:** Strategies of the ERAS program for TKA

Patients’ education
Inform detailed treatment process and rehabilitation plan
Continue knee joint functional exercise every hour during the day
Nutritional support and diet managements
Take high-protein dietary for at least 2 days before surgery
Individualized diet management on day of surgery: individually preoperative fasting and water-deprivation according to the operation time, oral carbohydrate solution 2 h before surgery, and early postoperative feeding on day of surgery.
General anesthesia
Restrictive fluid management
Intravenous crystalloid fluid volume controls in about 1500 ml on day of surgery
Minimally invasive operation
A middle skin incision, a standard medial parapatellar approach, and a measured resection technique.
Blood managements
Erythropoietin and chalybeate were used in patients with anemia (hemoglobin: male < 130 g/L; female < 120 g/L)
Controlled hypotension (mean arterial pressure: 70 to 80 mmHg) during operation
Tranexamic acid (TXA) utilization: a dose of 20 mg/kg intravenous TXA (IV-TXA) was given before incision, another two doses of 10 mg/kg or 1 g IV-TXA 3 and 6 h later.
Pain managements
Preventive analgesia with celecoxib orally preoperatively
Local infiltration of 80 mL 2.5% ropivacaine before incision closure
A standard regimen of oral nonsteroidal anti-inflammatory drugs (NSAIDs) after surgery, oral oxycodone as needed.
No allogenic blood transfusion
No tourniquet during operation
No urine catheter before surgery, catheter would be used for postoperative uroschesis.
No drainage tube or early removal of drainage tube after surgery
Antithrombotic prophylaxis
Low-molecular-weight heparin daily, starting 6 h postoperatively and given until discharge, then oral rivaroxaban 5 or 10 mg daily for 10 days.
Prevention of postoperative nausea and vomiting
Oral mosapride 5 mg 2 h before surgery and postoperatively three times a day
Dexamethasone 10 mg intravenous injection during operation and a repeat dose was given 4 to 6 h after surgery
Start rehabilitation and mobilization on day of surgery
Perform ankle flexion and extension exercises in bed immediately after surgery, then progressive ambulation exercises with full weight bearing on the first or second postoperative day with the assistance of a walker or crutches.
Discharge criteria
Be self-dependent, able to walk with crutches or better, able to achieve 100° active knee flexion or better and − 5° to 0° extension.

### Study outcomes

The potential variables associated with PLOS including preoperative patient characteristics, perioperative surgical factors, and postoperative complications were all collected during the inpatient hospital stay. Patient demographics of age, gender, BMI, diagnosis, comorbidities, ASA score, and preoperative knee function including flexion range of motion (ROM), varus or valgus deformity, and hospital for special surgery (HSS) knee score were recorded. Visual analogue scale (VAS) pain score, the serum level of hemoglobin (Hb), albumin, C-reactive protein (CRP), and interleukin-6 (IL-6) were recorded preoperatively and on postoperative days 1 and 3 (POD1, POD3). Perioperative surgical factors included operative time, surgery start time, intravenous fluid volume on day of surgery, total blood loss calculated by gross equation [13], utilization of catheter and drainage tube, and time to ambulate and achieve 90° active knee flexion after surgery. Postoperative complications including wound complications and other minor complications and preoperative LOS were also compared in two groups.

### Statistical analysis

Results were summarized using the mean and standard deviation for continuous variables, and counts and percentages for categorical variables. Continuous variables were tested for normality using the Kolmogorov-Smirnov test. Variables determined to be normally distributed were compared using Student’s *t* test, while variables non-normally distributed were compared using the Kruskal-Wallis non-parametric test. The relationship between each categorical variable and PLOS > 3 days was assessed using the chi-square test. If any expected or observed value was less than or equal to 5, Fisher’s exact test was used. All statistical tests were two-sided, and a *P* value of ≤ 0.05 was considered statistically significant. Following univariable analyses, significant factors were subjected to a stepwise multivariable logistic regression analysis to determine variables associated with prolonged PLOS using a *P* value ≤ 0.05 to remain in the model. Odds ratios (OR), corresponding 95% confidence intervals (CI), and *P* values were computed. IBM SPSS version 19.0 was used for all analyses.

## Results

Two-hundred forty-one consecutive primary TKA patients were included in the study (18.3% male, 81.7% female). The mean age was 65.9 years (range 38–89 years) while the mean BMI was 25.5 kg/m^2^ (range 14.5–35.9 kg/m^2^). Preoperative diagnosis was osteoarthritis (94.2%) or rheumatoid arthritis of the knee (5.8%). The patient characteristics and preoperative, perioperative, and postoperative variables of the study cohort are outlined in Table 2.

**Table 2:** The patient characteristics and preoperative, perioperative and postoperative variables by PLOS of patients underwent TKA (*N* = 241)

Variable	PLOS ≤ 3	PLOS > 3	*P* value
*N*	123	118	
Age (years)	65.5 ± 8.3	66.4 ± 8.5	0.374
Gender			0.856
Male	23 (18.7%)	21 (17.8%)	
Female	100 (81.3%)	97 (82.2%)	
BMI (kg/m^2^)	25.5 ± 3.5	25.5 ± 3.7	0.866
Diagnosis			0.027
Osteoarthritis	120 (97.6%)	107 (90.7%)	
Rheumatoid arthritis	3 (2.4%)	11 (9.3%)	
Comorbidities			
Hypertension	79 (64.2%)	74 (62.7%)	0.807
Diabetes	17 (13.8%)	20 (16.9%)	0.501
Heart disease	12 (9.8%)	11 (9.3%)	0.909
Pulmonary infection	7 (5.7%)	22 (18.6%)	0.002
ASA score			0.003
≤ 2	112 (91.1%)	91 (77.1%)	
3/4	11 (8.9%)	27 (22.9%)	
Preoperative knee function			
Flexion ROM (degrees)	105.2 ± 12.2	98.6 ± 17.9	0.001
Varus deformity	72 (58.5%)	66 (55.9%)	0.683
Valgus deformity	5 (4.1%)	19 (16.1%)	0.002
HSS score	50.9 ± 9.6	48.0 ± 8.2	0.014
VAS pain score			
Preoperatively	4.8 ± 0.8	5.1 ± 0.9	0.007
POD1	1.8 ± 0.7	1.9 ± 0.8	0.307
POD3	1.1 ± 0.4	1.3 ± 0.6	< 0.001
Hb (g/L)			
Preoperatively	129.4 ± 14.0	130.6 ± 13.5	0.478
POD1	117.4 ± 12.9	117.1 ± 12.4	0.879
POD3	109.4 ± 11.2	108.2 ± 11.9	0.408
Albumin (g/L)			
Preoperatively	43.0 ± 3.1	42.4 ± 3.4	0.186
POD1	38.9 ± 2.9	38.6 ± 3.1	0.378
POD3	36.4 ± 2.6	36.2 ± 3.4	0.674
CRP (mg/L)			
Preoperatively	3.4 ± 2.7	4.4 ± 5.0	0.070
POD1	23.6 ± 17.2	29.8 ± 20.6	0.011
POD3	41.8 ± 30.6	63.1 ± 44.7	< 0.001
IL-6 (ng/L)			
Preoperatively	5.2 ± 5.4	4.5 ± 3.0	0.219
POD1	33.1 ± 29.0	53.6 ± 42.1	< 0.001
POD3	25.9 ± 22.8	33.5 ± 27.9	0.021
Operative time (min)	80.1 ± 17.2	80.2 ± 15.2	0.982
Surgical start time			0.001
Before 2 PM	105 (85.4%)	80 (67.8%)	
After 2 PM	18 (14.6%)	38 (32.2%)	
Intravenous fluid volume (ml)	1464.5 ± 433.7	1533.1 ± 495.6	0.253
Total blood loss (ml)	595.0 ± 341.0	685.8 ± 373.6	0.050
Utilization of catheter	13 (10.6%)	28 (23.7%)	0.007
Utilization of drainage tube	48 (39.0%)	63 (53.4%)	0.025
Time to ambulate (day after surgery)	0.9 ± 0.4	1.2 ± 0.6	< 0.001
Time to achieve 90° active knee flexion (day after surgery)	1.5 ± 0.6	1.9 ± 0.8	< 0.001
Wound complications	3 (2.4%)	27 (22.9%)	< 0.001
Other minor complications	8 (6.5%)	39 (33.1%)	< 0.001
Preoperative LOS (day)	4.8 ± 1.4	5.6 ± 2.6	0.006

*PLOS* postoperative length of stay, *BMI* body mass index, *ASA score* American Society of Anesthesiologists score, *ROM* range of motion, *HSS score* hospital for special surgery knee score, *VAS* visual analogue scale, *POD1* postoperative day 1, *POD3* postoperative day 3, *Hb* hemoglobin, *CRP* C-reactive protein, *IL-6* interleukin-6; total blood loss, calculated by gross equation, *LOS* length of stay

The mean PLOS was 3.8 days (range 2–9 days) with a median of 3.0 days. There were 123 (51.0%) patients who had a PLOS ≤ 3 days and 118 (49.0%) a PLOS > 3 days. Table 2 outlines the univariable relationship of each variable with PLOS. Univariable analyses revealed a significant independent association between PLOS and diagnosis, preoperative pulmonary infection, ASA score, preoperative flexion ROM, valgus deformity, HSS score of the knee, VAS pain score preoperatively and on POD3, serum level of CRP and IL-6 on POD1 and 3, surgical start time, total blood loss, utilization of catheter and drainage tube, time to ambulate and achieve 90° active knee flexion after surgery, and wound complications and other minor complications. However, after multivariable adjustment, only preoperative valgus deformity of the knee (OR 4.95, 95%CI 1.56–15.77, *P* = 0.007), increased serum level of IL-6 on POD1 (OR 1.01, 95%CI 1.00–1.03, *P* = 0.039), increased VAS pain score on POD3 (OR 2.56, 95%CI 1.28–5.13, *P* = 0.008), increased serum level of CRP on POD3 (OR 1.01, 95%CI 1.00–1.03, *P* = 0.019), increased day to achieve 90° active knee flexion after surgery (OR 2.19, 95%CI 1.27–3.79, *P* = 0.005), wound complications (OR 8.58, 95%CI 2.10–35.03, *P* = 0.003), and other minor complications (OR 6.04, 95%CI 2.40–15.19, *P* < 0.001) remained significantly associated with prolonged PLOS in the final multivariable logistic regression model (Table 3).

**Table 3:** Multivariable logistic regression model for factors of PLOS > 3 days

Factor	Odds ratio	95%CI	*P* value
Preoperative valgus deformity of the knee	4.95	1.56–15.77	0.007
Increased serum level of IL-6 on POD1	1.01	1.00–1.03	0.039
Increased VAS pain score on POD3	2.56	1.28–5.13	0.008
Increased serum level of CRP on POD3	1.01	1.00–1.03	0.019
Increased day to achieve 90° active knee flexion after Surgery	2.19	1.27–3.79	0.005
Wound complications	8.58	2.10–35.03	0.003
Other minor complications	6.04	2.40–15.19	< 0.001

*PLOS* postoperative length of stay, *IL-6* interleukin-6, *POD1* postoperative day 1, *VAS* visual analogue scale, *POD3* postoperative day 3, *CRP* C-reactive protein

Thirty patients (12.4%) developed postoperative wound complications after surgery mainly including swelling (53.3%) and exudation (43.3%). Forty-seven patients (19.5%) developed other minor complications, which were predominately narcotic-related adverse drug events such as postoperative nausea and vomiting (PONV, 42.6%), fever (23.4%), hypotension (12.8%), and confusion (8.5%). Eleven patients (4.6%) developed both wound and other minor complications. Preoperative pulmonary infection (OR 2.75, 95%CI 1.20–6.28, *P* = 0.016), ASA score 3/4 (OR 2.14, 95%CI 1.01–4.52, *P* = 0.046), and utilization of catheter after surgery (OR 2.53, 95%CI 1.23–5.19, *P* = 0.012) were significantly associated with postoperative complications (Table 4).

**Table 4:** Multivariable logistic regression model for risk factors of postoperative complications

Factor	Odds ratio	95%CI	*P* value
Preoperative pulmonary infection	2.75	1.20–6.28	0.016
ASA score 3/4	2.14	1.01–4.52	0.046
Utilization of catheter after surgery	2.53	1.23–5.19	0.012

*ASA score* American Society of Anesthesiologists score

## Discussion

The most important finding of the present study was that preoperative valgus deformity of the knee, increased serum level of IL-6 on POD1, increased VAS pain score and serum level of CRP on POD3, increased day to achieve 90° active knee flexion after surgery, and postoperative wound complications and other minor complications were associated with prolonged PLOS of TKA. Meanwhile, preoperative pulmonary infection, ASA score 3/4, and utilization of catheter after surgery were statistically significant contributors in postoperative complications. This is a complete and systematic study in which preoperative, perioperative, and postoperative variables were all recorded and analyzed in patients who underwent TKA with the ERAS program. Moreover, factors such as preoperative valgus deformity of the knee and perioperative laboratory examination and pain level were investigated for the first time.

In the present study, the ERAS program focuses on the improvement of surgical techniques and optimization of perioperative management, which mainly contains preoperative patients’ education, nutritional support, minimally invasive operation, perioperative blood management, multimodal pain management, prevention of infection, venous thromboembolism and PONV, optimizing the use of drainage tube, catheter and tourniquet, and functional exercise. After implementation of the ERAS program, the patients who underwent TKA in our hospital could be discharged home on POD3. This study design may explain some of the discrepancies between previous studies without ERAS program and ours. In particular, we did not find an association between PLOS and age [5, 7], gender [5, 7], or BMI [4]; undeniably, this difference may be related to the characteristics of population.

Several studies have explored the association between comorbidities and PLOS [5–7, 14]. Winemaker et al. [14] reported that pre-existing cardiac comorbidities and diabetes treated orally were strong predictors of prolonged PLOS. However, the existence of heart disease or diabetes preoperatively differs from the control level of the diseases, which explain the reverse finding in the current study to some extent. ASA score, which is indicative of comorbidity, has been demonstrated to be predictive of prolonged hospital stay [5, 6]. Some preoperative comorbidities have been established to be predictive complications which are in turn linked to prolonged PLOS [5]. This is in accordance with our finding that patients with an ASA score 3/4 and preoperative pulmonary infection were 2.14 and 2.75 times more likely to have a postoperative stay > 3 days.

In the present study, preoperative knee function was associated with prolonged PLOS. This is in accordance with other studies [3, 15] on PLOS after TKA, with ERAS program as well as without ERAS program. Moreover, in addition to find that preoperative valgus deformity of the knee was an independent risk factor of prolonged PLOS, we observed that as the day to achieve 90° active knee flexion after surgery increases, the patients were more likely to have a prolonged PLOS. Previous studies rarely show these two variables as risk factors for PLOS, but instead they identified preoperative ROM of the knee or the use of walking aids [3, 15]. Mobilization as early as 4 to 6 h after surgery can help achieve functional recovery, reduce hospital stays, and improve functional outcomes [16].

Halawi et al. [17] retrospectively reviewed 112 consecutive patients who underwent total hip arthroplasty and found a significant association between PLOS and preoperative pain level. In this study, we analyzed the relationship between VAS pain score preoperatively and on POD1, POD3, and PLOS of TKA. Interestingly, we found that increased VAS pain score on POD3 was significantly associated with prolonged PLOS while preoperative VAS pain score was not. Postoperative pain remains an important barrier to early discharge of TKAs, and it has a major influence on the ability of the patient to resume normal activities at home [18]. Effective analgesia in the immediate postoperative phase is important to allow the patient to exercise and regain mobility, facilitating recovery while decreasing the PLOS.

Reducing surgical trauma is a target of TKA with the ERAS program; this could be evaluated by postoperative inflammation makers such as CRP and IL-6 level in serum. As Windisch et al. [19] reported, CRP increased within a few hours after TKA, with a maximum value between postoperative day 2 and POD3, then falling again to the normal level. This is consistent with the finding by Xie et al. [20] in which IL-6 level reached maximum on POD1. Therefore, we investigated the relationship between serum level of CRP and IL-6 on POD1, POD3, and PLOS of TKA. After a stepwise multivariable logistic regression analysis, we found that increased serum level of CRP on POD3 and increased serum level of IL-6 on POD1 were risk factors of prolonged PLOS. This is essential to monitor CRP and IL-6 in serum after TKA, although they were not strong predictors.

Postoperative complication as a risk factor for prolonged PLOS had been found in previous studies [7, 11, 21, 22]. However, in these studies, few had mentioned specific complications [7] or rarely investigated entire postoperative adverse events [11]. In our study, we found that patients who sustained wound complications or other minor complications were 8.58 and 6.04 times more likely to have a postoperative stay > 3 days compared to patients with no complication. Swelling and exudation were mainly wound complications, while other minor complications mainly included PONV, fever, hypotension, and confusion. These finding were consistent with findings in the literature [11, 22]. Furthermore, our study also found that preoperative pulmonary infection, ASA score 3/4, and utilization of catheter after surgery were significantly associated with postoperative complications in the multivariable logistic regression model. Therefore, every effort should be done to make further optimization on the patient’s medical comorbidities and reduce the utilization of catheter after surgery in order to have more favorable outcomes following TKA and decrease postoperative complications as well as PLOS.

There are several limitations to our study worth mentioning. Firstly, it was a retrospective chart review from a single tertiary-care teaching institution, while a prospective study might have provided us with additional information to predict the PLOS better. Moreover, the sample was relatively small in that a valid conclusion regarding age, gender, and BMI could not be made. Secondly, the need for blood transfusion may influence the PLOS of TKA [12, 23]. In the present study, this was not taken into account, since there was no patient who needed blood transfusion. Thirdly, we did not investigate the relationship between discharge destination and PLOS, because of that, every patient could be discharged home if they reached the discharge criteria in our hospital. Lastly, 30- and 90-day readmission was not assessed in the present study. Ricciardi et al. [24] demonstrated that short PLOS was a risk factor for 30- and 90-day readmission after TKA, while Sutton et al. [25] thought that early discharge was not an independent risk factor for 30-day major complications or readmissions following TKA. Therefore, further research should investigate whether early discharge influences the readmission rate of TKA with the ERAS program.

## Conclusions

In conclusion, preoperative valgus deformity of the knee, increased serum level of IL-6 on POD1, increased VAS pain score, and serum level of CRP on POD3, increased day to achieve 90° active knee flexion after surgery, and postoperative wound complications and other minor complications were associated with prolonged PLOS in the ERAS program for primary TKA. Preoperative pulmonary infection, ASA score 3/4, and utilization of catheter after surgery were statistically significant contributors in postoperative complications. It is important to recognize all the factors that associated with prolonged PLOS to try to maximize the use of medical resources and ultimately optimize the care of our patients.

## Abbreviations

ASA: American Society of Anesthesiologists
BMI: Body mass index
CI: Confidence intervals
CRP: C-reactive protein
ERAS: Enhanced recovery after surgery
Hb: Hemoglobin
HSS: Hospital for special surgery
IL-6: Interleukin-6
IV-TXA: Intravenous TXA
LOS: Length of stay
NSAIDs: Nonsteroidal anti-inflammatory drugs
OR: Odds ratios
PLOS: Postoperative LOS
POD1: Postoperative day 1
POD3: Postoperative day 3
PONV: Postoperative nausea and vomiting
ROM: Range of motion
TKA: Total knee arthroplasty
TXA: Tranexamic acid
VAS: Visual analogue scale
